# A Real-Time Smooth Weighted Data Fusion Algorithm for Greenhouse Sensing Based on Wireless Sensor Networks

**DOI:** 10.3390/s17112555

**Published:** 2017-11-06

**Authors:** Tengyue Zou, Yuanxia Wang, Mengyi Wang, Shouying Lin

**Affiliations:** College of Mechanical and Electronic Engineering, Fujian Agriculture and Forestry University, Fuzhou 350002, China; fafuwxm@163.com (Y.W.); yzzdyxlll@sina.com (M.W.); linshouying@fafu.edu.cn (S.L.)

**Keywords:** greenhouse, wireless sensor network, data fusion, dynamic weight

## Abstract

Wireless sensor networks are widely used to acquire environmental parameters to support agricultural production. However, data variation and noise caused by actuators often produce complex measurement conditions. These factors can lead to nonconformity in reporting samples from different nodes and cause errors when making a final decision. Data fusion is well suited to reduce the influence of actuator-based noise and improve automation accuracy. A key step is to identify the sensor nodes disturbed by actuator noise and reduce their degree of participation in the data fusion results. A smoothing value is introduced and a searching method based on Prim’s algorithm is designed to help obtain stable sensing data. A voting mechanism with dynamic weights is then proposed to obtain the data fusion result. The dynamic weighting process can sharply reduce the influence of actuator noise in data fusion and gradually condition the data to normal levels over time. To shorten the data fusion time in large networks, an acceleration method with prediction is also presented to reduce the data collection time. A real-time system is implemented on STMicroelectronics STM32F103 and NORDIC nRF24L01 platforms and the experimental results verify the improvement provided by these new algorithms.

## 1. Introduction

In recent years, wireless sensor networks (WSNs) have been widely used to monitor the environment [1,2,3], such as the temperature, humidity, gas concentration, gas composition, dust and so on, particularly for agricultural production purposes [4,5,6]. A greenhouse is an agricultural facility designed to extend the production season and improve the quality of agricultural products [7]. Because greenhouses generally contain climate-regulating equipment, the internal sensing data of greenhouses must be comprehensive and accurate. WSNs are composed of hundreds or thousands of sensor nodes that are used to acquire parameters under a range of conditions and transmit these parameters to a base station or sink node [8,9,10], enabling an information-based decision to be made in an automated manner. They are often used as the system for fire alarm in forests or used to monitor the environment in the field. In a greenhouse, the number of sensor nodes may be less. But if the greenhouse is large, it may also need several hundreds of sensor nodes. Furthermore, in complex and inhomogeneous environments, noise often corrupts the sensing data. Thus, a data fusion algorithm is required for selecting correct reports from mass data to identify accurate values from the measurements [11,12,13]. 

**Figure 1:**
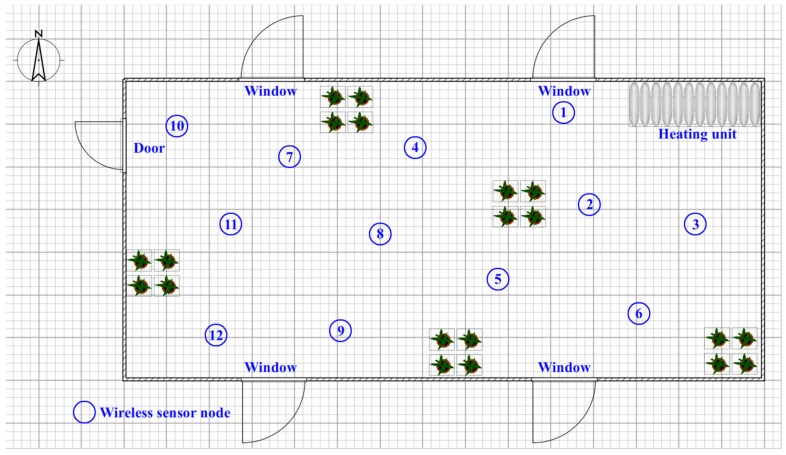
Illustration of a sensor network deployed in a greenhouse.

Figure 1 shows an illustration of a sensor network deployed in a greenhouse containing a heating unit and windows. When the heating unit is working, the temperature of the area nearest to the unit may become hotter than other regions far from the heater because the heating effect is related to the distance from the actuator. This effect can be termed the ‘actuator effect’ and tends to disrupt sensor reports by introducing inhomogeneous information into the dataset. The actuator influence is common in agricultural and industrial environments; for example, when sunlight shines through windows in the roof of a greenhouse, the light intensity value detected in small regions increases but most parts of the greenhouse remain dark. If the automatic control system follows the high light intensity value, subsequent incorrect operation is challenging to avoid. Thus, a data fusion algorithm is expected to reduce the influence of the actuator effect or other disturbing factors during the sensing procedure; the subsequent analysis of correct data is meaningful for the automation of the agriculture industry.

To improve the accuracy of sensing and reduce the burden of data transmission, researchers are now focusing on data fusion mechanisms [14,15,16]. A data fusion scheme based on a grey model and an extreme learning machine has been proposed to reduce redundant transmissions and extend the lifetime of the network [17]. The performance of this technique has been demonstrated through simulation. The Peeling algorithm [18] was developed to improve the performance of serial data fusion and the simulation results highlight the effectiveness of this algorithm in reducing energy consumption and time responsiveness. A distributed data fusion algorithm, which aims to minimize energy cost, was also deployed in the active network paradigm using WSNs [19]. The optimal linear estimation method was also derived to achieve data fusion in multi-rate sensor networks [20]. The fusion of quantized and un-quantized sensor data has been investigated to improve estimates [21]. Task-oriented distributed fusion functions have been introduced to adapt the dynamics of tasks and the topology of self-organized networks [22]. Rough-set and back-propagation neural networks have also been adopted to raise the accuracy of prediction [23]. Maximum a priori probability [24] and vector-space-based methods [25] can be used to reduce the influence of noisy or conflicting data in sample values. Neural networks have also been used to assist the data fusion for sensing [26,27]. The neural networks can help to select important features from datasets and improve the fusion work. They can also be used to optimize the localization for WSNs [28]. Feature selection and integration are also important techniques for data fusion. The adaptive weight and feedback control can help to find out the key points from the datasets efficiently [29]. However, few researchers have addressed the inhomogeneous effect of environmental elements influencing the sampled data of WSNs. Furthermore, most of the studies typically prove new algorithms through simulation only and not through real-time hardware and experimentation. Thus, this study focuses on the solution of noisy data produced by the inhomogeneous effect in certain application circumstances and the mechanism to speed up calculations. An embedded hardware platform is built to evaluate the effectiveness of these new algorithms and simulations are run on a large-scale dataset.

The contributions of this study are as follows: (1) An adjacent graph is introduced to describe the regional relationship among sensor nodes. The value assigned to the arc of the graph is used to indicate the distance between two neighboring nodes; (2) A stable area-searching method based on Prim’s algorithm is designed to locate regions that are not influenced by the actuator effect; (3) A voting mechanism based on dynamic weight is introduced to complete the data fusion and the self-healing function is designed to distinguish the actuator effect from normal sensing; (4) An acceleration mechanism with adaptive threshold is proposed to speed up the process of decision making in real-time applications.

The remainder of this paper is organized as follows: Section 2 presents a detection method for stable areas and the data fusion procedure based on a voting mechanism. In Section 3, an acceleration method is designed to speed up the computational process in real-time applications. Experiments are performed on embedded hardware and the results are presented in Section 4. Finally, the conclusions are provided in Section 5.

## 2. Data Fusion and Self-Learning

To improve the accuracy of the final decision, a data fusion mechanism is required to reduce the influence of an actuator. The actuator effect is related to the position under a certain circumstance. Once the location of a sensor node is set, the accuracy of the node’s sensing data is approximately confirmed. The improved method must detect the nodes influenced by the actuator and reduce their contribution via a weighting method in the data fusion algorithm.

### 2.1. Adjacent Graph and Stable Area

**Figure 2:**
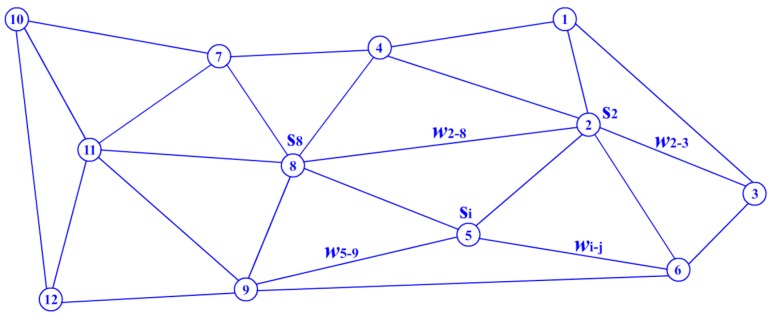
Illustration of an adjacent graph for a sensor network.

To find the stable area in the map, the spatial relationships of the sensor nodes should first be determined. An adjacent graph is used in this work to describe the regional relationship between neighboring nodes. The adjacent graph is a type of undirected graph that uses arc connections to express the relationship between neighboring nodes. As shown in Figure 2, a node in the adjacent map represents a sensor node whose location is recorded by the engineer manually or using its locating device. A weight value *w_i–j_* (*i* < *j*) is assigned to each arc according to the distance between node *i* and *j*, which is normalized as shown in Equation (1).

(1) $$w_{\mathrm{i–j}}=\frac{w_{\mathrm{i–j-org}}-w_{\min}}{w_{\max}-w_{\min}}$$

where *w_i–j-org_* represents the original straight-line distance between sensor node *i* and *j*, *w*_min_ denotes the minimum distance between two sensor nodes in the sensor network and *w*_max_ is the maximum value.

Furthermore, a smoothing value *S_k_* is calculated for each node in the graph following Equations (2) and (3) to express the uniformity of sensing data in the node’s neighborhood. The actuator effect is gradually reduced as the distance from actuator device increases. Thus, along with the spreading paths of the effect, the sensing data in that region is not uniform. This key point can help determine the main scope of the actuator’s effect in the map.

(2) $$s_{\mathrm{k-org}}=\sum_{i=1}^{t}\left(D_{i}-D_{k}\right)/t$$

(3) $$s_{k}=\frac{s_{\mathrm{k-org}}-s_{\min\mathrm{-org}}}{s_{\max\mathrm{-org}}-s_{\min\mathrm{-org}}}$$

where *t* is the number of neighboring nodes for the center node *k* according to the adjacent graph; *D_k_* represents the data acquired from node *k* and *D_i_* is the data from its neighboring node *i*; *s_k-org_* denotes the original smoothing value for node *k*; and *s*_min-_*_org_* and *s*_max-_*_org_* are the minimum and maximum smoothing values, respectively.

After the construction of the adjacent graph, the weight of each arc *w_i–j_* and the smoothing value *S_k_* for every node are generated. As Algorithm 1 shows, a method based on Prim’s algorithm is introduced in this work to search a stable area for data fusion. Prim’s algorithm is a classical approach to compute a minimum spanning tree from a graph and a smoothing value threshold *λ* is set to avoid using nodes in unstable areas. Thus, the stable area can be marked by the node set *U* in the result; the time complexity of the algorithm is O(|V|^2^). Using only the sensing data from stable areas, the final fusion decision can be made and the result is recorded as history that can influence future decision making.

**Algorithm 1.** Procedure to find a stable area via a search algorithm**Input:**The adjacent graph ({*V*, *S*, *E*, *W*}), in which *V* = {*u*_0_, *u*_1_, *u*_2_, …} is the set of nodes with corresponding *S* = {*s*_0_, *s*_1_, *s*_2_, …} as their smoothing values and *E* is the set of arcs with *W* as their weights.
**Output**: a set of nodes *U* in the stable area**Start:**Sort the set *V* from small to large by their smoothing value *S* to make *V’*Initialize *U* = {*u*_0_}, *O* = {} and *TE* = {}, in which *u*_0_ is the first vertex in *V’*, *O* is a set for storing obsolete vertexes during operation and *TE* is a set for storing valid arcs during operation.**while**
*U*∪*O* ≠ *V’*    Find the arc (*u_i_*, *v_j_*) with minimum weight that satisfies *u_i_*∈*U*, *v_j_*∈*V’-U-O*    **if**
*s_j_* < *λ* (*s_j_* is the smoothing value for vertex *v_j_*)           *U* = *U*∪{*v_j_*}; *TE* = *TE*∪{(*u_i_*, *v_j_*)}    **else**           *O* = *O*∪{*v_j_*}    **end if****end while****end**

### 2.2. Data Fusion Based on Dynamic Weight

After acquiring the stable area, the data fusion result can be generated according to that area. Because the reports are not the same in all applications, a principle should be designed to determine the extent to which each node participates in the final decision. The voting mechanism based on weight is a satisfactory choice for this principle. Equation (4) shows the procedure of voting, where $\hat{Y}$ denotes the data fusion result, *ϒ_i_* represents the report value from sensor node *i*, *ω_ti_* is the corresponding time-related weight for node *i* at time *t* and *s_i_* is the corresponding smoothing value.

(4) $$\hat{Y}=\sum_{i=1}^{N}\omega_{ti}s_{i}{ϒ}_{i}/\sum_{i=1}^{N}\omega_{ti}s_{i}$$

**Figure 3:**
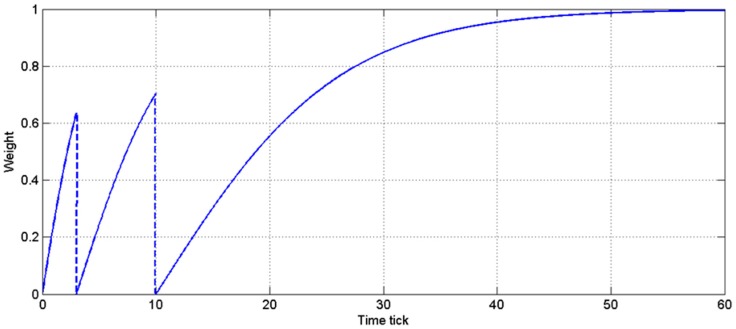
Example of the weight of a sensor node influenced by the actuator.

When an actuator works, low weights should be assigned for the sensor nodes near it, whereas normal weights should be used when the actuator is closed. Because of the challenges associated with continually describing the working time of an actuator, a dynamic weight with self-healing ability is proposed. Equation (5) shows the rule that determines the dynamic weight, where *n* indicates the continuous count of being outside the stable area, *α* is an adjustment parameter for the variation control and *t* denotes the time ticks after the setting process. When the stable area is confirmed at a new time tick, the weights of the sensor nodes outside the area are refreshed by Equation (5). For example, if a sensor node is outside the stable area at a time tick, this node’s weight follows the curve of 1–2/(1 + e*^αt^*). Furthermore, if the node is outside the stable area again before its weight recovers to 1, the curve should be refreshed by the function of 1−2/(1 + e^0.5×*αt*^); otherwise, the curve follows the previous curve until the node’s weight is restored to value 1. Thus, based on this mechanism, the sensor nodes adjacent to the actuator can gradually recover participation in data fusion over time to recover from the actuator effect. Continuous regions that remain outside the stable area enhance the effect of the decreased weight and the discontinuous regions do not influence the data significantly. Figure 3 shows the weight variation of a sensor node adjacent to an actuator in the application. The node enters the unstable area at time ticks 0, 3 and 10 and its weight is gradually reduced to avoid the actuator’s influence.

(5) $$\omega_{ti}=1-\frac{2}{1+e^{\frac{1}{2^{n-1}}\cdot \alpha \cdot t}}$$

## 3. Acceleration Mechanism with an Adaptive Threshold

Since the data fusion mechanism is now influenced by the voting procedure, the precision of the result can be improved. The conventional voting procedure nevertheless requires that data are collected from all participants. This approach can lead to an increase in time latency for automatic decision and can become an obstacle to real-time application. To minimize the time consumption for decision making, an acceleration mechanism is proposed in this section to achieve a balance between accuracy and speed.

### 3.1. Procedure of the Acceleration Mechanism

**Figure 4:**
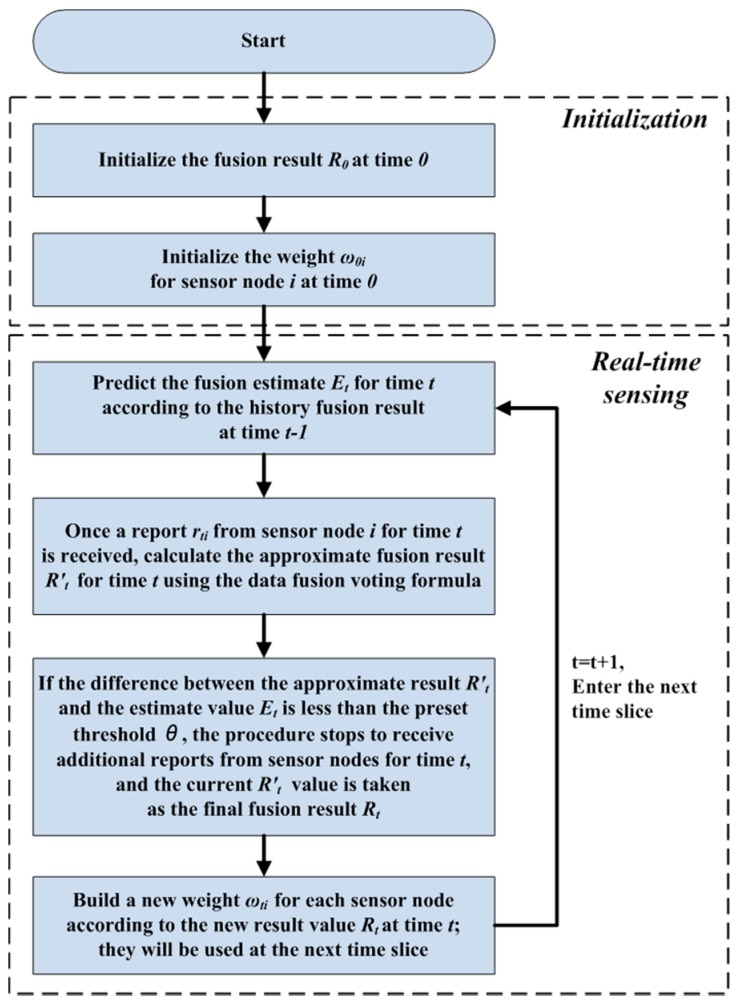
Procedure of the acceleration mechanism.

According to the voting algorithm, the workstation must wait until all data, from all sensor nodes, are collected to make a final decision. However, communicational obstacles may exist for data transmission in large sensor networks or networks with faulty nodes that exhaust their energy or encounter problems with the corresponding equipment. In these extreme cases, the data fusion program must wait for the data from each node until timeout, which slows down data collection and increases the response time of the system in real-time applications. Thus, an acceleration mechanism with adaptive thresholds is designed to address this problem. As shown in Figure 4, the acceleration is based primarily on a forward prediction and a backward adaptive threshold adjustment. The whole procedure can be divided into two steps, the initialization and real-time sensing. At the initialization step, the value of the data fusion result at time 0 and the weighting for each sensor node are assigned according to the voting algorithm. Furthermore, at the real-time sensing step, the workstation starts to receive reports from sensor nodes and refreshes the fusion result. In circumstances with uniform parameters, only some of the reports from the sensor nodes (rather than all of them) are required due to the similarity of the reports. 

Thus, the procedure generally uses the history value at time *t* − 1 to estimate a value for time *t* via a prediction algorithm before the formal data fusion occurs. Then, once the workstation receives a report *r_ti_* from sensor node *i* for time *t*, an approximate fusion result *R'_t_* is calculated for time *t* using the data fusion voting formula. Then, if the difference between the approximate result *R'_t_* and the estimate value *E_t_* is less than the pre-set threshold *θ*, the procedure stops to receive additional reports from sensor nodes for time *t* and the current *R'_t_* value is taken as the final fusion result *R_t_*. After that, the weight of each sensor node is refreshed according to the new result value *R_t_* at time *t* and is used for the next time *t* + 1. Following this design, under the circumstance of uniform parameters that change linearly, the acceleration algorithm soon converges to a final decision with little error. However, under complex environments, the algorithm requires additional time to obtain a relatively accurate result. The balance between accuracy and speed is controlled by the prediction method and the pre-set threshold *θ*, whose large value allows for more error but uses less time to acquire a result. Equations (6) and (7) show the adaptive threshold recommended in this work. As the variation of measuring object increases, *θ* decreases to enhance restriction for data fusion, which may avoid larger errors in the result. Furthermore, intermittent use of acceleration mechanisms can also help maintain the balance between accuracy and speed. For example, after the acceleration procedure is used three times, we omit using it the fourth time, using instead the result calculated from all the reports to help eliminate the accumulated error.

(6) $$\Delta x_{k}=\left|x_{k}-x_{k-1}\right|$$

(7) $$\theta =E+\varepsilon \cdot (1/\sqrt{2\pi }\sigma )e^{-\frac{{\left(\Delta x_{k}-\mu \right)}^{2}}{2\sigma^{2}}}$$

where Δ*x_k_* represents the variation of measuring object from time *k* − 1 to time *k*; *E* is a fixed value assigned by the engineer and *ε* is an adjustment value selected from a Gaussian distribution. 

### 3.2. Prediction Algorithm

The prediction algorithm is another important element for the acceleration mechanism. The physical parameters measured by sensor networks are regulated linearly or non-linearly and the prediction algorithm should be designed for various parameters with this factor in mind. This research focuses on the most general environmental parameters for agriculture, such as temperature, humidity, light and CO_2_ concentration. Because these parameters do not typically change sharply, a Kalman filter is a suitable choice for prediction.

The purpose of the Kalman filter is to minimize the squared error of the estimated non-stationary signal in the noise. Each state update in the Kalman filter is recursively calculated from its previous estimate and the latest input data so that it is necessary to store only the previous estimate without all the past observations. Thus, the Kalman filter is widely used to reduce the influence of sensing and processing noise in applications that predict subsequent values. We consider the linear system described by the state space and measuring space:

(8) $$\{\begin{array}{c} x(k)=A(k-1)x(k-1)+B(k)n(k)\\ y(k)=C^{T}(k)x(k-1)+D(k)n_{1}(k) \end{array}$$

where *x*(*k*) represents the (N + 1) × 1-dimensional state variable vector; *y*(*k*) is an observation signal vector; and *n*(*k*) and *n*_1_(*k*) denote the process noise and observation noise, respectively. If *M* is the number of system inputs and L is the number of system outputs, then *A*(*k* − 1), *B*(*k*),*C*(*k*) and *D*(*k*) are coefficient matrixes with dimensions (N + 1) × (N + 1), (N + 1) × M, (N + 1) × L and L × L, respectively.

Let $\hat{x}(k|k-1)$ represent the estimated value of *x*(*k*) using the observation value until time *k* − 1 and let $\hat{x}(k|k)$ denote the estimated value of *x*(*k*) using the observation value until time *k*. Thus, the corresponding estimated error can be defined as Equations (9) and (10). Furthermore, the corresponding covariance matrix of these errors can be calculated using Equations (11) and (12).

(9) $$e(k|k)=x(k)-\hat{x}(k|k)$$

(10) $$e(k|k-1)=x(k)-\hat{x}(k|k-1)$$

(11) $$R_{e}(k|k)=E\left[e(k|k)e^{T}(k|k)\right]$$

(12) $$R_{e}(k|k-1)=E\left[e(k|k-1)e^{T}(k|k-1)\right]$$

(13) $$R_{n}(k)=E\left[n(k)n^{T}(k)\right]$$

(14) $$R_{n_{1}}(k)=E\left[n_{1}(k)n_{1}{}^{T}(k)\right]$$

Algorithm 2 shows the procedure for using the Kalman filter, where *K*(*k*) is an (N + 1) × L matrix called the Kalman gain and $R_{n}(k)$ and $R_{n_{1}}(k)$ are defined in Equations (13) and (14), respectively. Using the $\hat{x}(k|k-1)$ as the prediction value for time *k*, the acceleration mechanism can be performed according to the adaptive threshold *θ*.

**Algorithm 2.** Kalman filter procedure**Initialization:** $\hat{x}(0|0)=x(0)\;$$R_{e}(0|0)=x(0)x^{T}(0)$
**Calculation:** when *k* ≥ 0,  $\hat{x}(k|k-1)=A(k-1)\hat{x}(k-1|k-1)$
  $R_{e}(k|k-1)=A(k-1)R_{e}(k-1|k-1)A^{T}(k-1)+B(k)R_{n}(k)B^{T}(k)$
  $K(k)=R_{e}(k|k-1)C(k){\left[C^{T}(k)R_{e}(k|k-1)C(k)+R_{n_{1}}(k)\right]}^{-1}$
  $\hat{x}(k|k)=\hat{x}(k|k-1)+K(k)(y(k)-C^{T}(k)\hat{x}(k|k-1))$
  $R_{e}(k|k)=\left[I-K(k)C^{T}(k)\right]R_{e}(k|k-1)$


## 4. Simulation and Experiments

### 4.1. Simulation Results and Discussion

**Figure 5:**
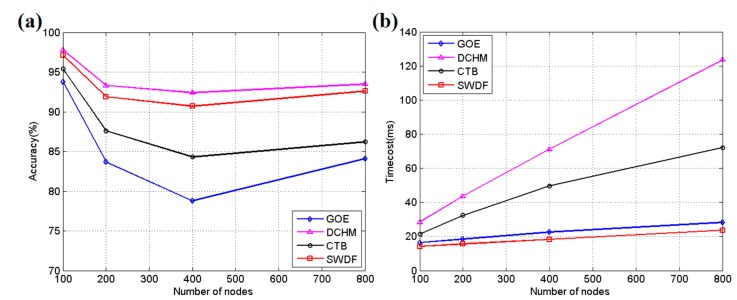
(**a**) The average accuracy for methods in the simulation; (**b**) The average time/cost for the methods in the simulation.

To verify the effect of the smooth weighted data fusion (SWDF) process introduced in this work, a simulation implemented in MATLAB is performed on a PC with a 3.4 GHz Intel Core CPU and 4 GB memory. In the simulation, the sensor nodes are uniformly deployed in a 400 m × 400 m rectangular field and 20 actuators are randomly settled in the field with an influencing range consisting of a 5 m radius circle. Four different algorithms are involved in the simulation: the GM-OP-ELM (GOE) method [17], double cluster head model (DCHM) [14], a cosine theorem-based method for identifying and fusing conflicting data (CTB) [25] and the SWDF introduced in this paper. GOE uses a grey model and an extreme learning machine to predict the data of the next period to accelerate the calculation process. Moreover, the DCHM selects two cluster heads in each group and adopts Bayesian data fusion to improve robustness. In CTB, a fusion algorithm based on the degree of mutual support is proposed to accommodate conflicting data. The parameters were set to *ε* = 0.17 in GOE and *α* = 1, *λ* = 0.3, *E* = 0.3, *μ* = 0, *σ* = 1 and *ε* = 1 in SWDF. The ratio of compromised sensor nodes is set to 30% in the DCHM. Figure 5 shows the simulation results for 100, 200, 400 and 800 sensor nodes when sensing the environmental temperature. These sensor nodes are uniformly deployed in the simulation field. Three hundred simulation tests are run for each group and the average results are calculated for presentation. 

As shown in Figure 5a, the DCHM obtained the highest average accuracy in these four algorithms. The accuracy represents the degree of sensing data following the real one, as calculated by Equation (15). The accuracy decreases as the density of the sensor nodes increases, indicating that the actuator’s effect is gradually increasing. When the number of nodes reaches 800, the accuracy exhibits a small rebound due to the influence of multiple nodes outside the influencing range of actuators. We found that the DCHM, CTB and SWDF all featured capabilities to reduce the influence of the actuator’s effect on the simulation, while GOE did not obtain satisfactory performance because it lacked a robust way to accommodate distorted data. The DCHM, consisting of a weighted DBSCAN (Density-Based spatial clustering of applications with noise) algorithm [14], Bayesian data fusion and double cluster heads mechanism, is found to be a powerful approach to address actuator disturbance.

(15) $$\left(1-\frac{\left|d_{s}-d_{r}\right|}{d_{r}}\right)\times 100\%$$

where *d_s_* represent the sensing value acquired by the WSN system and *d_r_* denotes the real value.

**Figure 6:**
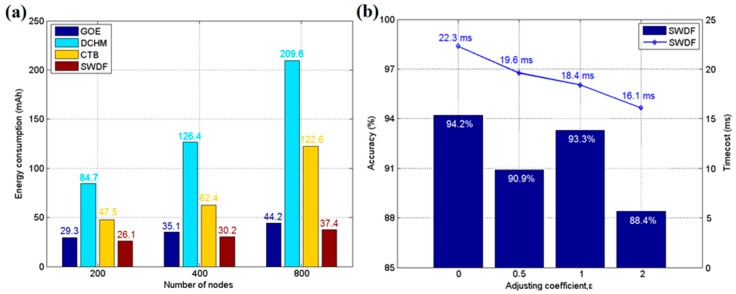
(**a**) The average energy consumption of each sensor node over an hour; (**b**) The average accuracy and time/cost for various adjustment coefficients *ε*.

However, the large calculation burden of the DCHM, as shown in Figure 5b, makes it challenging to deploy in real-time applications, particularly when the devices of the applications are equipped with a low-frequency MCU. Figure 5b shows the time/cost of each round of sensing calculations for these methods. GOE and SWDF are designed with a prediction-based accelerating method, which does not require the reports from all nodes to make a final decision. GOE and SWDF enable a low time/cost when the number of sensor nodes increases, while other algorithms do not efficiently control the time consumption. The DCHM requires the most calculation time in each group, due primarily to the cluster head re-selection procedure. In WSNs, the energy is an important factor of interest to engineers because sensor nodes often contain limited battery capacity that is not easily recharged. Figure 6a shows the average energy consumption in an hour for each testing group. SWDF is well suited for low-energy-consumption applications in real-time circumstances relative to other traditional methods, particularly in agricultural applications using low-cost hardware.

The process of parameter setting is another area of concern in SWDF application. With fewer parameters, the SWDF is simple and sufficiently efficient to be used over a large-range sensing area. The adjustment coefficient *ε* in Equation (7) determines the value of the adaptive threshold *θ*, which influences the balance between accuracy and speed. When *α* = 1, *λ* = 0.3, *E* = 0.3, *μ* = 0 and *σ* = 1 and *ε* is set to 0, 0.5, 1, or 2, Figure 6b shows the effect of these various *ε* values in SWDF working with 200 sensor nodes. A different *ε* may lead to a different degree of involvement of the adaptive part of the tolerant threshold for prediction error. When *ε* = 0, the threshold is fixed and it costs 22.3 ms for each sensing round. As *ε* increases, the adaptive part has more effect in Equation (7) and leads to a different sensing time and accuracy. As shown in Figure 6b, the influence of *ε* is not monotonic. When *ε* = 1, the accuracy is larger than when *ε* = 0.5. Thus, this value should be assigned by the user according to the application environment. Brief on-site testing is recommended to determine a suitable *ε* value after the algorithm is deployed. A satisfactory choice of this value will lead to quick response of the system and (in some cases) high accuracy.

### 4.2. Implementation and Experiments

**Figure 7:**
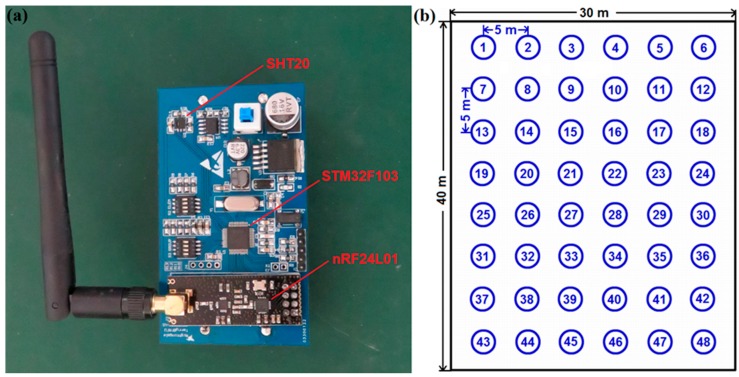
(**a**) Illustration of the wireless sensor node for experiments; (**b**) Illustration of the deployment of sensor nodes for experiments.

**Figure 8:**
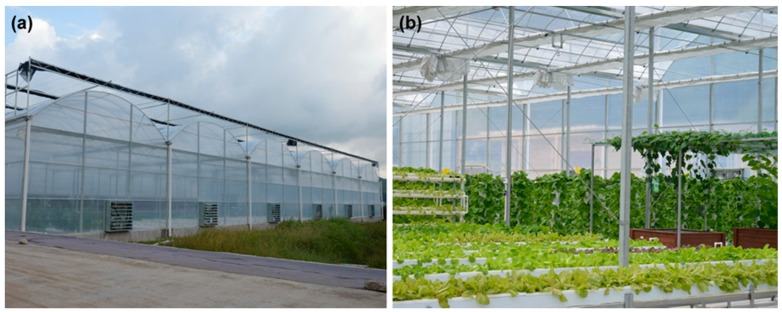
(**a**) Illustration of the appearance of the experimental greenhouse; (**b**) Illustration of the inside structure for the experimental greenhouse.

As shown in Figure 7a, to further verify the effect of the SWDF in real-time applications, the hardware of a WSN node was implemented on STMicroelectronics STM32F103 and NORDIC nRF24L01 platforms with a SHT20 temperature and humidity sensor from Sensirion Corporation. The STM32F103 is an ARM 32-bit Cortex-M3 microcontroller that is used to drive the sensor and execute calculations. The nRF24L01 is a single-chip 2.4 GHz transceiver with an embedded baseband protocol engine, which is used to establish the WSN in the sensing system. The SHT20 is a humidity and temperature sensor with fully calibrated digital output. Each chip should be calibrated by the producer before it comes to the market. Thus, it is not necessary to calibrate the sensor nodes before our experiments. The hardware is established on FR-4 PCB board with lithium battery for providing power. As Figure 8 shows, the experiments were performed in a 30 m × 40 m rectangular greenhouse containing 12 fans, shutters, outside shades and inside shades as actuators. The 48 sensor nodes were arranged in a line at 5 m intervals as shown in Figure 7b. The SWDF, DCHM and the general average algorithm (GAA), without any data fusion mechanisms, were evaluated in the experiments. The parameters for SWDF in experiments were set to *α* = 1, *λ* = 0.3, *E* = 0.3, *μ* = 0, *σ* = 1 and *ε* = 1 and the ratio of compromised sensor nodes in DCHM was set to 30%. 

**Figure 9:**
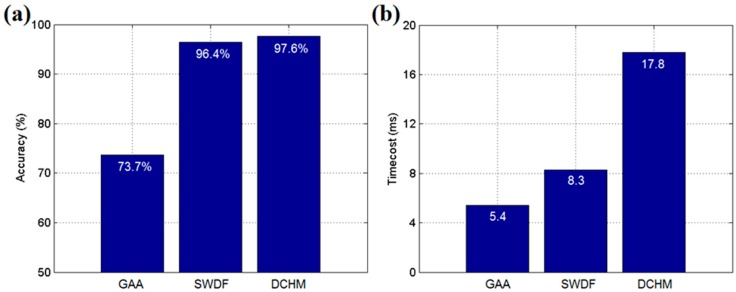
(**a**) Average accuracy of each algorithm in the experiments; (**b**) Average time cost of each algorithm in the experiments.

The real temperature values for comparison in experiments were calculated using specialized sensor nodes in the correct area of the greenhouse, as chosen manually. A correct area means a robust area for data sensing. The sensor nodes inside the correct area will never be influenced by actuators and therefore they will not report data imprecisely. The correct area can be found automatically by previous experiments. If the error between the sensing data is less than the set variance, the sensor node can be considered to be located within the correct area. Furthermore, the detection boundary of the outermost correct nodes can be considered as the boundary of the correct area. The specialized sensor nodes are generally chosen manually from correct area which can provide stable and precise parameter values for experiments. One way for choosing these specialized nodes is to select the nodes far away from the actuators on the map and ensure that they will not be affected by any actuator; another way is to observe the nodes over a period of time in previous experiments and find out the nodes with stable sensing data. Only several specialized sensor nodes are sufficient and the average value of their sensing data will be regarded as the estimated real temperature value in the environment. The experiments were run for ten rounds and at each round, a refreshment of 10.8 thousand data bytes were acquired for each algorithm. The temperature refreshing time was set to once per second and each round lasted 3 h. The experimental results for these three algorithms are shown in Figure 9.

As shown in Figure 9, the accuracy of SWDF was similar to that of the DCHM in experiments. However, the GAA obtained the lowest accuracy because none of the data fusion mechanisms were deployed; thus, the average value of all of the reports from the sensor nodes was taken as the result. Moreover, the DCHM cost more than twice the time taken by SWDF and GAA used the least time/cost of the three algorithms. In summary, we found experimentally that SWDF is an efficient method that can use limited time to obtain a stable data fusion result. The SWDF algorithm, deployed in real-time applications, will work particularly well for the agricultural WSN with a low cost of hardware and multiple sensor nodes. Although the SWDF is not necessary for a greenhouse control, it can improve the robustness of the sensing system. Because the operation of the environmental control system in the greenhouse depends largely on the correctness of the sensing data, the SWDF can help to avoid the malfunction and overshoot of the actuator. That is very meaningful for greenhouse production. This algorithm can also be used in the fields outdoor but the effect is not as significant as in a closed greenhouse.

## 5. Conclusions

Accurate sensor results are important for automatic control in agricultural and industrial applications. However, sensor nodes near actuators may encounter noisy data. To increase the system robustness, we propose the SWDF method, in which voting data fusion (based on adjacent graph theory) is designed to reduce the influence of data corruption and dynamic weights are proposed to maintain a balance between accuracy and speed. To shorten the response time, an acceleration mechanism with a prediction algorithm is developed to reduce the number of redundancy reports from sensor nodes. A real-time hardware system is implemented on the STM32F103 MCU and nRF24L01 wireless platforms. Simulations and experiments in a greenhouse demonstrate the improvements achieved using the new set of algorithms.

## References

[1] Kampianakis E., Kimionis J., Tountas K., Konstantopoulos C., Koutroulis E., Bletsas A. (2014). Wireless environmental sensor networking with analog scatter radio and timer principles. IEEE Sens. J..

[2] Iqbal M., Naeem M., Anpalagan A., Ahmed A., Azam M. (2015). Wireless sensor network optimization: Multi-objective paradigm. Sensors.

[3] Zou T., Lin S., Feng Q., Chen Y. (2016). Energy-Efficient Control with Harvesting Predictions for Solar-Powered Wireless Sensor Networks. Sensors.

[4] Polo J., Hornero G., Duijneveld C., García A., Casas O. (2015). Design of a low-cost Wireless Sensor Network with UAV mobile node for agricultural applications. Comp. Electron. Agric..

[5] Jiang J.A., Wang C.H., Liao M.S., Zheng X.Y., Liu J.H., Chuang C.L., Hung C.L., Chen C.P. (2016). A wireless sensor network-based monitoring system with dynamic convergecast tree algorithm for precision cultivation management in orchid greenhouses. Precis. Agric..

[6] Xu L. (2015). Design of a RSSI location system for greenhouse environment. Int. J. Distrib. Sens. Netw..

[7] Li T., Zhang M., Ji Y., Sha S., Jiang Y., Li M. (2015). Management of CO_2_ in a tomato greenhouse using WSN and BPNN techniques. Int. J. Agric. Biol. Eng..

[8] Lai Y., Xie J., Lin Z., Wang T., Liao M. (2015). Adaptive data gathering in mobile sensor networks using speedy mobile elements. Sensors.

[9] Cayirpunar O., Kadioglu-Urtis E., Tavli B. (2015). Optimal base station mobility patterns for wireless sensor network lifetime maximization. IEEE Sens. J..

[10] Yin X., Fang D., Wang W., Chen X. (2016). EETC: To transmit or not to transmit in mobile wireless sensor networks. Wirel. Netw..

[11] Moshou D., Pantazi X.E., Kateris D., Gravalos I. (2014). Water stress detection based on optical multisensor fusion with a least squares support vector machine classifier. Biosyst. Eng..

[12] Moshou D., Gravalos I., Bravo D.K.C., Oberti R., West J.S., Ramon H. (2011). Multisensor fusion of remote sensing data for crop disease detection. Geospatial Techniques for Managing Environmental Resources.

[13] Felisberto F., Fdez-Riverola F., Pereira A. (2014). A ubiquitous and low-cost solution for movement monitoring and accident detection based on sensor fusion. Sensors.

[14] Fu J.S., Liu Y. (2015). Double cluster heads model for secure and accurate data fusion in wireless sensor networks. Sensors.

[15] Palafox-Albarran J., Jedermann R., Hong B., Lang W. (2015). Cokriging for cross-attribute fusion in sensor networks. Inf. Fusion.

[16] Soganli A., Ercetin O., Cetin M. (2015). On the Quality and Timeliness of Fusion in a Random Access Sensor Network. IEEE Signal Process. Lett..

[17] Luo X., Chang X. (2015). A novel data fusion scheme using grey model and extreme learning machine in wireless sensor networks. Int. J. Control Autom. Syst..

[18] Mostefaoui A., Boukerche A., Merzoug M.A., Melkemi M. (2015). A scalable approach for serial data fusion in Wireless Sensor Networks. Comp. Netw..

[19] Lu Z., Tan S.L., Biswas J. (2013). Fusion function placement for Active Networks paradigm in wireless sensor networks. Wirel. Netw..

[20] Yan L., Jiang L., Xia Y., Fu M. (2016). State estimation and data fusion for multirate sensor networks. Int. J. Adapt. Control Signal Process..

[21] Saska D., Blum R.S., Kaplan L. (2015). Fusion of Quantized and Unquantized Sensor Data for Estimation. IEEE Signal Process. Lett..

[22] He H., Zhu Z., Mäkinen E. (2015). Task-oriented distributed data fusion in autonomous wireless sensor networks. Soft Comp..

[23] Rawat S., Rawat S. (2016). Multi-sensor data fusion by a hybrid methodology-A comparative study. Comp. Ind..

[24] Ferrari G., Martalò M., Abrardo A. (2014). Information fusion in wireless sensor networks with source correlation. Inf. Fusion.

[25] Zhang Z., Liu T., Chen D., Zhang W. (2014). Novel algorithm for identifying and fusing conflicting data in wireless sensor networks. Sensors.

[26] Jing L., Wang T., Zhao M., Wang P. (2017). An Adaptive Multi-Sensor Data Fusion Method Based on Deep Convolutional Neural Networks for Fault Diagnosis of Planetary Gearbox. Sensors.

[27] Si L., Wang Z., Liu X., Tan C., Xu J., Zheng K. (2015). Multi-sensor data fusion identification for shearer cutting conditions based on parallel quasi-newton neural networks and the Dempster-Shafer theory. Sensors.

[28] Chuang P.J., Jiang Y.J. (2014). Effective neural network-based node localization scheme for wireless sensor networks. IET Wirel. Sens. Syst..

[29] Tian G.Y., Gledhill D. Visualisation based feedback control for multiple sensor fusion. Proceedings of the Tenth International Conference on Information Visualisation (IV’06).

